# Preventive health examinations: protocol for a prospective cross-sectional study of German employees aged 45 to 59 years (Ü45-check)

**DOI:** 10.3389/fpubh.2023.1076565

**Published:** 2023-06-12

**Authors:** Linda Kalski, Franziska Greiß, Johannes J. Hartung, Lorena Hafermann, Maja A. Hofmann, Bernd Wolfarth

**Affiliations:** ^1^Institute of Sport Science, Humboldt-Universität zu Berlin, Berlin, Germany; ^2^Department of Sports Medicine, Charité – Universitätsmedizin Berlin, Berlin, Germany; ^3^Institute of Biometry and Clinical Epidemiology, Charité – Universitätsmedizin Berlin, corporate member of Freie Universität Berlin and Humboldt-Universität zu Berlin, Berlin, Germany; ^4^Department of Dermatology and Venereology, Charité – Universitätsmedizin Berlin, Berlin, Germany; ^5^Federal German Pension Insurance Berlin-Brandenburg, Berlin, Germany

**Keywords:** screening, prevention, rehabilitation, medical health examinations, disability pension, Ü45-check

## Abstract

**Objective:**

Early identification of health-related risk factors is of great importance for maintaining workability. Screening examinations can help to detect diseases at an early stage and provide more needs-based recommendations. This study aims (1) to assess the individual need for prevention or rehabilitation based on preventive health examinations compared to a questionnaire survey, (2) to assess the results of the preventive health examinations compared to the Risk Index – Disability Pension (RI-DP), (3) to assess the results of the questionnaire survey compared to the RI-DP, (4) to assess the general health status of the sample (target population > 1,000) in German employees aged 45–59, (5) to identify the most common medical conditions. A further study question aims, and (6) to investigate the general health status of the specific occupational groups.

**Methods:**

Comprehensive diagnostics including medical examination, anamnesis, anthropometric measurements, bioelectrical impedance analysis (BIA), handgrip strength, resting electrocardiogram (ECG), resting blood pressure, pulse wave velocity (PWV), and laboratory blood analyses added by a questionnaire are conducted. The research questions are analyzed in an exploratory manner.

**Results and conclusion:**

We expect that the results will allow us to formulate recommendations regarding screening for prevention and rehabilitation needs on a more evidence-based level.

**Clinical Trial Registration**: DRKS ID: DRKS00030982.

## Introduction

Demographic change is a topic that is not only being discussed in Germany today but has been known for decades. High-birth-rate cohorts such as the baby boomers will retire or take early retirement in the near future. The shrinking population in Germany and the demographic aging are increasing challenges for the healthcare system and the social insurance system (1). The COVID-19 pandemic may have led to a rise in early labor cessation in the baby boomer generation, which represents an additional challenge (2).

Various diseases can lead to incapacity for work. Some of the risk factors can expand unnoticed over decades, namely an unhealthy diet, smoking, excessive alcohol consumption, and a lack of physical activity. Overweight and obesity numbers have risen in industrialized countries in recent years (3). Due to the high number of risk factors, specific prevention and rehabilitation measures should be developed to prevent and reduce diseases (4). Screening examinations are encouraged to determine an individual’s risk of developing diseases. In order to find out which program or therapy is individually appropriate, health checks should be performed. It is important that public healthcare institutions provide information about screening examinations and ensure access (5). The focus should be on education regarding the early detection of diseases so that individuals are able to evaluate the benefits and participate in screening examinations. It is important to target asymptomatic individuals to detect possible diseases at an early stage (6, 7) as non-communicable diseases, like cardiovascular diseases, are the leading cause of mortality globally (8) and account for 40% of all deaths (9). Screening examinations could assess the risk of developing cardiovascular disease in Germany (6).

For a feasible and effective screening, a comparison of different screening models is required. While screening examinations were once dismissed as unimportant because of their little yield, in recent years they have moved more into focus again. The reason for this is that screenings are being developed in a more targeted manner, such as precisely for pre-symptomatic individuals or certain age groups (10).

The study ‘Ü45-Check’ addresses interests and questions from the fields of politics, medicine, and science. In 2016, a pension law (11) was passed in Germany that allows a more flexible entry into retirement, following the Scandinavian model (12). Three political parties (Social Democratic Party of Germany (SPD), Alliance 90/The Greens (Bündnis 90/Die Grünen), and Free Democratic Party (FDP)) have formed a coalition in Germany in December 2021. In the coalition agreement for 2021–2025 (13), a paragraph on prevention and rehabilitation has been written, stating that healthier working should be the focus of pension policy. The associated principle ‘prevention before rehabilitation before retirement’ promises simplified access to prevention and rehabilitation programs. Following this, the present study is listed in the coalition agreement for 2021–2025 on page 58 (13).

The challenge of simplifying access to prevention and rehabilitation programs is of particular interest to public health reducing sick leave and disability pensions potentially leading to higher costs for the social insurance systems, such as the German Pension Fund (GPF) (14, 15).

To gain further insight into the characteristics of disability pension applicants, a risk level prediction index was developed in a previous research project. Bethge et al., 2011 (16) developed the Risk Index – Disability Pension (RI-DP). They identified variables of prognostic relevance for disability pension in the register data of the GPF and constructed a risk index for work disability that is applied in the present study.

The primary aim of this study is to assess the need for prevention or rehabilitation based on preventive health examinations compared to a questionnaire survey by another research group (17, 18). As secondary aims, we, first of all, want to examine the influence on relationship between the medical examinations, the questionnaire, and the RI-DP on a disability pension. As further secondary aims, we are interested in assessing the general health status of the sample of German employees aged 45–59. Thereby, we are interested in the most common medical conditions, the health status of the specific occupational groups, and the need for rehabilitation.

### Primary study question

1. Are there differences in the assessment of the need for prevention or rehabilitation based on the preventive health examinations compared to accessing the need for preventive or rehabilitation measures by a questionnaire survey?

### Secondary study questions

2. Is there any relation between the results of the preventive health examinations and the RI-DP Index (traffic light systems)?
3. Is there any relation between the results of the questionnaire survey and the RI-DP Index (traffic light systems)?
4. What is the general health status of the sample of German employees aged 45–59?
5. Which diseases are most common in the sample?
6. What is the general health status of specific occupational groups?

## Methods/study design

### Study design and population

**Figure 1:**
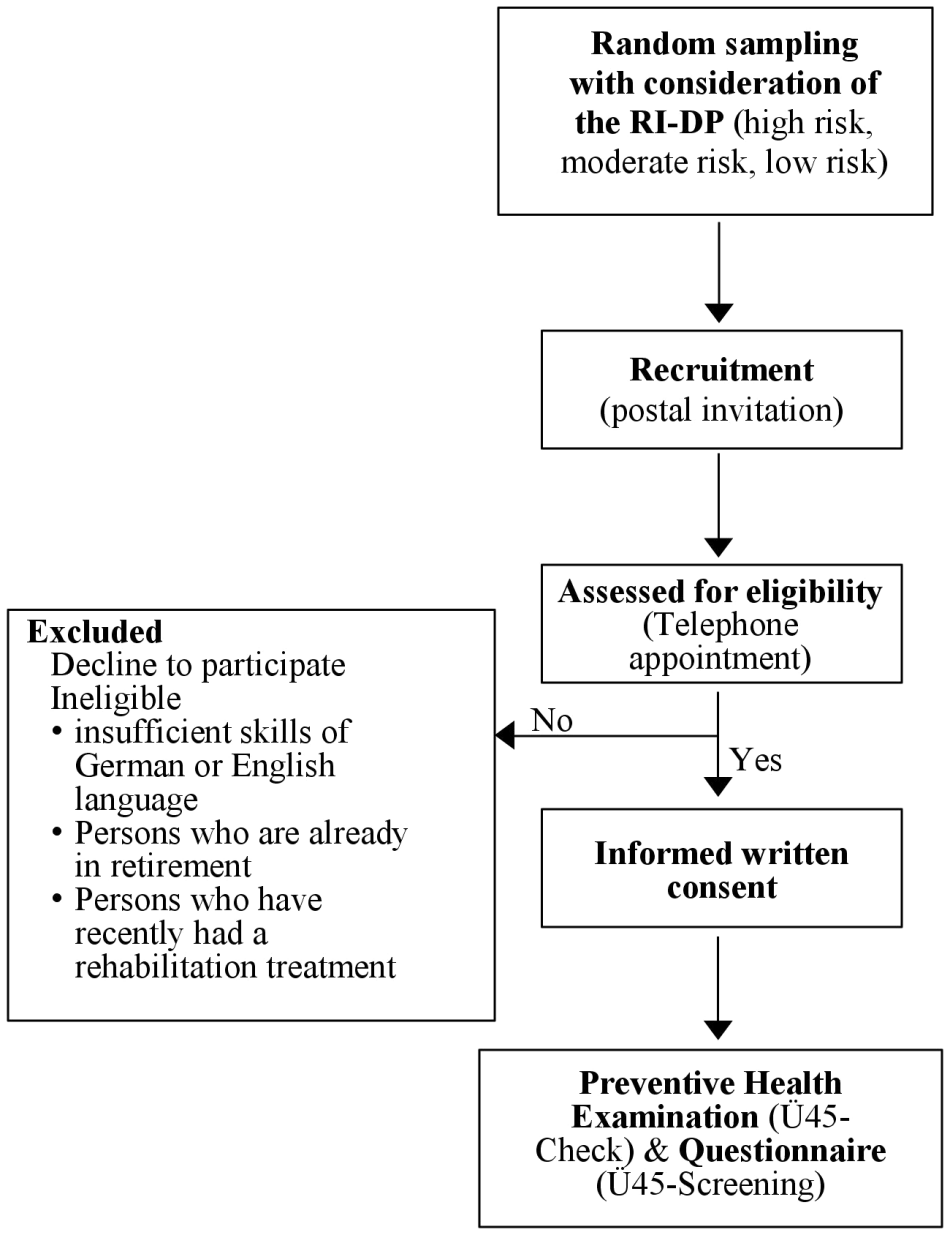
Study flow diagram, Ü45-Check; Germany 2022; Risk Index – Disability Pension (RI-DP).

This study is designed as a cross-sectional trial to investigate the implementation and evaluation of preventive health examinations offered to adults aged 45 to 59 years in Berlin and Brandenburg, Germany. Administratively, all persons insured by the GPF who were between 45 and 59 years old and living in Berlin or Brandenburg in June 2021 were identified in the GPF Registers and allocated to one of three groups in terms of risk for work disability [see *Risk Index – Disability Pension* (*RI-DP*)]. The subjects (target population > 1,000) will be randomly selected from each of the three groups and will be invited to attend the preventive health examination (Ü45-Check) in the following two consecutive calendar years (2021 to 2023) (Figure 1).

### Inclusion criteria

- Insured persons of GPF, residence in Berlin or Brandenburg
- Age of 45 to 59

### Exclusion criteria

- Insufficient skills in German language or English language
- Persons who are already in early retirement
- Persons who have recently had a rehabilitation treatment

### Recruitment and invitation

All subjects will receive a postal invitation from the GPF including information about the objectives of the Ü45-Check health examination. Subjects can voluntarily contact Charité – Universitätsmedizin Berlin by phone or email to schedule an appointment. A suggested appointment time is provided. The scheduled time may be accepted, modified, or rejected via phone or email. Three months after the postal invitation, the subjects receive a letter for the evaluation of the Ü45-Check. The evaluation is part of another research group.

### Risk index – Disability pension

The study of the RI-DP was designed as a case–control study of the GPF using data from 8,500 men and 8,405 women. Independent samples (control group) were used to validate their models. The information revealed by that index can be used to enhance the provision of rehabilitation programs. The RI-DP can be calculated based on secondary data for the 3 years preceding the reference year. A person has a high risk of disability pension if the RI-DP is ≥60, a moderate risk is <60 und ≥ 50, and a low risk is <50 (16, 19).

In our study, the RI-DP is collected and considered during recruitment. It does not influence the study participation or non-participation. Physicians and study participants do not have any information about the respective index value (of the subject), only the project management does.

### Setting

The preventive health examinations are performed in the outpatient clinic of the Department of Sports Medicine, Charité – Universitätsmedizin Berlin/Humboldt-Universität zu Berlin. Charité – Universitätsmedizin operates as an independent contractor within the public health service and is remunerated based on a combination of fee-for-service.

### Questionnaire (Ü45-screening)

Before the health examination, subjects are asked to answer a web-based questionnaire, which may be completed in approximately 10 min. The questionnaire was developed by another research group in 2019, as part of the Ü45-Check (17, 18). The questionnaire includes five dimensions adapted from already established questionnaires, which have been proven to help identifying an already existing limitation of the ability to work or predict a hazard. The questions relate to dimensions of workability, mental health, functional ability, coping behavior, and sports and exercise behavior (Table 1). Items were reused from the Work Ability Index (WAI) (20), SIMBO (21), PHQ-4 (22), IRES-3 (23), and General Practice Physical Activity Questionnaire (GPPAQ) (24). The questionnaire can be found in Supplement S1. Subjects are informed about the opportunity for responding to the questionnaire together with study staff if support is needed for any reason.

**Table 1:** Ü45-Screening questionnaire.

	Measurement	Technical information
Questionnaire	**Ü45-Screening** Work ability Mental health Functional ability Coping behavior Sports and exercise behavior	IPAD 6th generation, Apple, U.S.

The questionnaire is evaluated using a point system, accordingly, different values depending on the answer to each question, and dimensions are evaluated separately and weighted differently. Further details can be found here (17, 18). Three domains are categorized in the questionnaire evaluation: no action needed, prevention program suggested, and rehabilitation program suggested. Accordingly, ‘need for action’ (e.g., rehabilitation program suggested in the dimension of ‘workability’) is present when the score reaches more than half of the possible points (≥ 7 of 12 possible points). Prevention program in the dimension of ‘work ability’ is recommended if the score reaches 1/3 of all possible points (≥ 4 points) (17, 18).

### Preventive health examination

The health professionals at Charité – Universitätsmedizin Berlin will perform the clinical examination. The examination in total takes approximately 120 min including the following diagnostics: Anthropometric measurements, bioelectrical impedance analysis (BIA), handgrip strength, systolic and diastolic blood pressure (SBP and DBP) (25), resting electrocardiogram (ECG), and pulse wave velocity (PWV) (26). The subjects will wear underwear during instrumental diagnostics.

Details about the diagnostics and measurements are given in Table 2.

**Table 2:** Measurements in preventive health examination Ü45-Check.

Clinical measures	Measurement	Technical information
Anthropometry	**Height (cm), Weight (kg)** Body-Mass-Index (BMI) (kg/m^2^)	Seca 274 Stadiometer, Seca, Germany
	**Waist (cm), Hip (cm)** Waist-to-Hip-Ratio (WHR) (Waist cm/Hip cm)	Seca 201 measuring band, Seca, Germany
Body composition	**Bioelectrical Impedance Analysis (BIA)** Percent body fat (PBF), Fat free mass (FFM), Visceral fat area (VFA), Total body water (TBW), Intracellular water (ICW), Extracellular water (ECW), Phase angle (PhA)	InBody 770, Inbody, South Korea
Handgrip strength diagnostics	**Handgrip Strength** (2 times each hand)	Hand dynamometer Lite, Baseline Evaluation Instruments, Germany
Cardiovascular diagnostics	**Twelve-lead Resting Electrocardiogram (ECG)**	Custo cardio 400, Custo med, Germany
Vascular diagnostics	**Blood Pressure (BP)** Systolic blood pressure (SBP) Diastolic blood pressure (DBP)	Classic III Stethoscope, 3 M Littmann, U.S.; boso med I, BOSCH + SOHN GmbH, Germany
	**Pulse Wave Velocity (PWV)** **Pulse Wave Analysis (PWA)**	Vicorder, SMT, Germany
Anamnesis	Chronic or current symptoms, medical history, family history, medication, dietary habits/supplements Cardiovascular risk factors, activity and training	IPAD 6th generation, Apple, U.S.
Cardiovascular examination	Cardiac examination Pulmonary examination Abdominal examination Peripheral vascular Examination	CORE Digital-Stethoscope, 3 M Littmann, U.S.
Blood sampling and analyses	**Blood Analyses** Blood cell and differential cells count, electrolytes, C-reactive protein (CRP), liver enzymes (GOT (AST), GPT (ALT), gamma-GT), blood lipid levels (total cholesterol, HDL-cholesterol, LDL-cholesterol and triglycerides), renal function parameters, ferritin, fasting glucose, Glycosylated hemoglobin (HbA1C), thyroid-stimulating hormone (TSH), urine test strip	

In the following consultation with a physician, a careful anamnesis regarding the relevant medical history is conducted. As most days missed at work are due to cardiovascular, orthopedic, or mental illness, former medical reports are screened for relevant cardiopulmonary, orthopedic, or psychosocial diseases or risk factors. Furthermore, medical needs and current health problems addressed, giving advice on further treatment and evaluation. In addition information about regular exercise/physical activity, health-relevant habits, as well as a healthy diet, is given.

The physical examination will focus on the cardiovascular system, lungs, and abdomen. The blood analyses will be taken under fasting conditions. These include screening parameters for diabetes, lipid profile, inflammatory markers as well as liver enzymes, and thyroid hormones. In addition, a urine sample is screened for proteinuria, hematuria, leukocyturia, and other abnormal parameters (Table 2).

### Assessment of body composition

A non-invasive bioelectrical impedance analysis (BIA) will be conducted to estimate body composition. Well-trained study staff will perform the measurement according to the standardized procedure. Subjects will be instructed to abstain from caffeine and alcohol for 24 h, and exercise for 12 h before testing according to published guidelines for BIA (27). Multiple frequencies at 5, 20, 250, and 500 kHz will be used to measure intracellular and extracellular water separately. The subjects will be measured under laboratory conditions standing barefoot, in underwear, and without wearing jewelry on the device. With abducted arms 15° and legs 45° apart, they will hold a hand electrode with a contact of all 10 fingers while heels and forefeet will be placed appropriately on the foot electrode. Then, an alternating current of 250 mA of intensity will be applied to measure the impedance of the arm, trunk and leg muscles. Whole-body resistance will be calculated as the sum of segmental resistance (right arm, left arm, trunk, right leg, left leg). The BIA with InBody 770 (Seoul, Korea) has been validated by dual-energy X-ray absorptiometry (28). In normal and overweight adults, multiple frequency BIA underestimated the percentage of body fat within the precision of the BIA instrument (2%) (27, 28).

### Assessment of handgrip strength diagnostics

The handgrip strength diagnostics is measured by a research assistant using a hand dynamometer (Baseline Evaluation Instruments, Germany) (29). Subjects are encouraged to squeeze a hand dynamometer as hard as possible using one hand. Handgrip strength is measured in a seated position with the elbow flexed at 90°, adjacent to the torso, and the thumb facing upward. Each hand is tested twice, alternating hands between trials with a 1-min rest between tests on the same hand (30).

### Assessment of (cardio) vascular diagnostics

A Twelve-lead resting electrocardiogram (ECG) (Custo Cardio 200 Saug-EKG, Custo med GmbH, Ottobrunn, Germany) is performed.

First, the blood pressure is measured on both arms using the device (Vicorder, SMT Medical Technology GmbH, Germany). Then the pulse wave analysis is performed on the right upper arm, while the cuff is inflated to the diastolic pressure. In preparation for the PWV examination, a standard cuff is placed at the upper thigh on the right leg. Subsequently, a special collar is placed at the lateral right side of the neck over the common carotid artery region.

### Traffic light system

**Figure 2:**
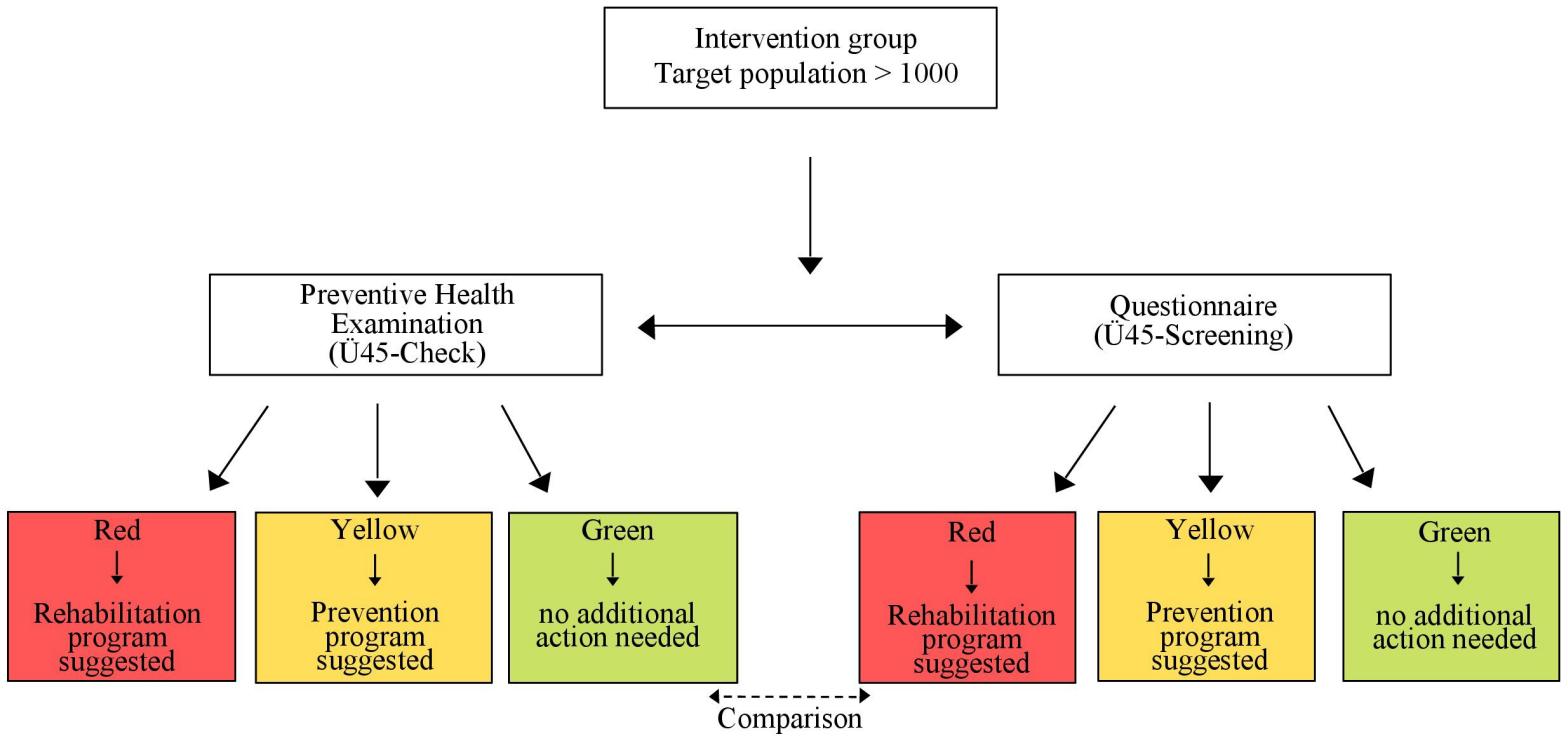
Study flow diagram, Ü45-Check Traffic light system; Germany 2022.

Immediately after the Ü45-Check, the subject’s health condition is assessed by physicians in a traffic light system. The study physician determines the health status based on the medical history and the examination results, but without knowing how the result of the questionnaire (Ü45-Screening) turned out. The subjects receive the results of each measurement and overall assessment of their health condition. Further examinations are needed and recommendations are offered stratified by the risk profile of the individual (Figure 2).

## Statistical methods

All analyses will be conducted in an explorative setting. In the first step, a descriptive analysis will be performed summarizing all variables with absolute and relative frequencies for categorical variables, and means and standard deviations for continuous variables. Based on the primary study question, the hypothesis is that the assessment based on preventive health examinations differs from the assessment according to the Ü45-questionnaire. Hence, for the primary endpoint, the score according to the Ü45-Screening questionnaire will be evaluated first. Afterward, the results of the classification of patients into green, yellow and red based on the Ü45-Screening questionnaire are compared with the classification according to the physician’s assessment. Therefore a Stuart-Maxwell test will be used to test for marginal homogeneity and a weighted kappa coefficient using linear weights will be calculated. In the second step, the differences between the results based on the Ü45-Screening questionnaire and the medical assessment will be visualized using boxplots and histograms for all variables based on the clinical examination and the questionnaire, respectively. In addition, these variables are used to build two ordinal regression models, one that uses the physician’s assessment as the dependent variable and one with the assessment by the Ü45-Screening questionnaire as the dependent variable. In each case, the green category is used as a reference. The two regression models are compared to determine the factors influencing the various ratings. Secondary aims (2) and (3), which aim to compare the assessment by means of the Ü45-Screening questionnaire and the medical assessment with the RI-DP index, respectively, will also be assessed by visualization for all variables using boxplots and histograms. Since the RI-DP index measures a different outcome from the assessment by the Ü45-Screening questionnaire and the medical assessment, a statistical test will not be used.

In order to assess the general health status of the sample, the variables of the anamnesis and the clinical examination are of main interest. Those variables are analyzed descriptively with absolute and relative frequencies for categorical variables, and means and standard deviations for continuous variables. In addition, this is also conducted as a subgroup analysis for different occupations. In order to assess which diseases are the most common in the sample, the absolute and relative frequencies of each disease are calculated and compared.

If missing values are present, their structure is analyzed and based on this, multiple imputation is considered.

### Ethics approval and consent to participate

Ethics approval was obtained by the “Ethics Committee of the Faculty of Culture, Social and Educational Sciences, Humboldt-Universität zu Berlin” on August 20, 2020 (reference number: HU-KSBF-EK_2020_0010). The work described is carried out in accordance with the Declaration of Helsinki for experiments involving humans. All subjects are informed by the study staff about the study procedure, subsequent data storage, and confidentiality and pseudonymity regarding the data. Written informed consent is collected from all subjects that the study center is allowed to use the data for research analyses and publishing the data.

### Trial registration and status

The trial was registered at the German Clinical Trials Register (DRKS-ID: DRKS00030982). Retrospectively registered December 27, 2022, https://drks.de/search/de/trial/DRKS00030982.

Recruitment of participants started at June 16, 2021 and will last until March 31, 2024. Until now (April 2023), 660 participants were successfully recruited and screened.

## Discussion

The outcomes of the Ü45-Check study comprise different aspects of health-related risk factors. The medical check-up can detect subclinical diseases and risk factors and reveal impairments ahead of time (5, 6, 31, 32). The Ü45-Check is based on available evidence on screening and preventive health examinations, closely aligned with the healthcare system in Germany. The study has been designed to develop a screening examination. The long-term goal of the federal government in Germany is to implement a screening examination in primary care. The evaluation of this screening method is part of another research project.

A screening program has the potential to label an asymptomatic person as a patient. Being labeled with a disease could have adverse effects, e.g., anxiety, worries, or panic attacks (33). Krogsbøll et al. (34) found in a meta-analysis that the majority of general health checks had no positive effect on morbidity, mortality and absence from work. A critical analysis reveals that the meta-analysis includes articles from the year 1963–1999 (34, 35). It is important to develop further studies and collect new data, considering demographic change and digitalization. Mortality as a parameter is not a sufficient criterion for the impact of health examinations, e.g., quality of life should be assessed (31). Since then, the working environment and leisure activities has changed completely. Obesity and the metabolic syndrome have increased over recent decades, which has become a growing and worldwide issue (36). Globally, social media screen time and game console screen time are on the rise. Living a sedentary lifestyle is a risk factor for various diseases.

There are also studies that have come to a different conclusion. A health check-up can have beneficial psychological and physical effects on the individuum. Health consciousness can be strengthened through a screening examination, which can lead to individuals being more motivated to be physically active and eat healthier (37). A healthier lifestyle leads to a better quality of life. Preventive measures that address participants’ personal health practices are beneficial and have an influence on the participant’s future health. Patients should be informed about health risks and be involved in the decision-making process of possible preventive measures (38). Studies related to prevention examinations have shown that the level of psychological stress induced by screening is short lasting (39).

Prevention and Rehabilitation programs can be cost-intensive in the short term, but they are beneficial to the healthcare system in long term by addressing diseases sooner. A European model study showed that a health check assessing vascular diseases would be cost-effective in the six countries included (Denmark, France, Germany, Italy, Poland, and the United Kingdom) (40).

Another aspect is that non-participation in screening examinations is a well-known problem (41). The problem is that the need for lifestyle intervention of people who do not participate in preventive screening cannot be identified. Furthermore, it can be assumed that subjects that participated in the study might have had medical needs and therefore replied to the invitation more often than healthy individuals did (6).

### Strengths

Early detection of risk factors is related to a better outcome (32, 42, 43) and can potentially prevent the manifestation of associated diseases or secondary complications. Not only is this important to prevent high costs for the social system, but we also suspect that the results of a comprehensive check-up had a health benefit for the individual (44).

The individual examinations of the Ü45-Check were carefully selected, on the one hand, to get a significance of the results and on the other hand that it can be carried out from an economic point of view in other institutions, like resident physicians. Key strengths of the Ü45-Check include the use of standardized methods to assess body composition and the use of previously validated measurement procedures [e.g., vascular diagnostics and handgrip strength diagnostics (26)]. Handgrip strength is well established as an indicator of muscular function, particularly among older adults (45). In this context, the measurement of handgrip strength is gaining importance as a screening tool. A low skeletal muscle mass leads to an impairment of physical functionality and quality of life (46). Several studies also showed an association between decreased handgrip strength and increased mortality and hospitalization rates, so it is already being used in geriatric assessment (46–48). Handgrip strength can be recommended as a screening for identifying patients at risk of poor health status (49, 50). Furthermore, the PWV is a marker of aortic stiffness and enables a noninvasive measurement and analysis of the cardiovascular system. The association with cardiovascular risk is well-established in adults (51, 52).

Another strength is that the sample size also allows for more extensive statistical analyses (such as the analysis of factors influencing the various assessments using an ordinal regression model).

### Limitations

Self-selection is connected to health consciousness, a state of being aware while willingly engaging in health-promoting activities, behavior, and lifestyle. A risk of bias could be that people who feel worse may have been more likely to accept the invitation for the Ü45-Check. However, it would also be possible that individuals with more risk factors may tend to choose not to attend preventive medical check-ups. The RI-DP could enable an active strategy to enhance participation in check-ups. The study design is a cross-sectional study, so it cannot be ascertained whether the participants accepted the recommendation for prevention or rehabilitation. However, a follow-up is planned and requested in the informed consent.

By considering the RI-DP in our study we have a selection bias. Therefore study question four, reflecting the general health status of the sample of German employees aged 45–59, cannot be answered for the general population.

## Conclusion

The results of the Ü45-Check program, which is being conducted on behalf of the federal government, will provide a scientific basis that is important for primary health care.

Collaboration among a variety of organizations/professionals within an organization is useful to ensure successful screening programs (53).

## Data availability statement

The original contributions presented in the study are included in the article/Supplementary material, further inquiries can be directed to the corresponding author.

## Ethics statement

The studies involving human participants were reviewed and approved by Ethics Committee of the Faculty of Culture, Social and Educational Sciences, Humboldt-Universität zu Berlin. The patients/participants provided their written informed consent to participate in this study.

## Author contributions

LK: funding acquisition, project administration, conceptualization, methodology, data collection, data curation, writing original draft. FG and JH: preventive health examination. LH: statistical support. MH: recruitment. BW: funding acquisition, conceptualization, and supervision. All authors contributed to the article and approved the submitted version.

## Funding

The study was funded by the GPF Berlin-Brandenburg. The sampling was carried out by the GPF Berlin-Brandenburg. The funder does not play a role in the interpretation of the data. The article processing charge was funded by the Deutsche Forschungsgemeinschaft (DFG, German Research Foundation) – 491192747 and the Open Access Publication Fund of Humboldt-Universität zu Berlin.

## Conflict of interest

The authors declare that the research was conducted in the absence of any commercial or financial relationships that could be construed as a potential conflict of interest.

## Publisher’s note

All claims expressed in this article are solely those of the authors and do not necessarily represent those of their affiliated organizations, or those of the publisher, the editors and the reviewers. Any product that may be evaluated in this article, or claim that may be made by its manufacturer, is not guaranteed or endorsed by the publisher.

## Abbreviations

BIA: Bioelectrical Impedance Analysis
BMI: Body-Mass-Index
DBP: Diastolic blood pressure
ECG: Electrocardiogram
GPF: German Pension Fund (German: Deutsche Rentenversicherung)
PWV: Pulse wave velocity
RI-DP: Risk Index – Disability Pension (German: RI-EMR = Risikoindex – Erwerbsminderungsrente)
SBP: Systolic blood pressure
WHR: Waist-to-Hip-Ratio
