# Supplementary material for: Preventive health examinations: protocol for a prospective cross-sectional study of German employees aged 45 to 59 years (Ü45-check)
Source: Front Public Health. 2023 Jun 12;11:1076565. doi: 10.3389/fpubh.2023.1076565 (PMC10291684; doi:10.3389/fpubh.2023.1076565)
Supplement: Supplementary file 1 [file Data_Sheet_1.PDF]

## Work Ability

1. How long were you on sick leave in the past 12 months?

☐ not at all

☐ 1 day to 6 weeks

☐ more than 6 weeks up to 3 months

☐ more than 3 months up to 6 months

☐ more than 6 months

2. Does any illness or impairment currently interfere with your work?
*Please tick everything that applies to you. Multiple answers are possible. If you are currently unemployed, refer to your last performed job.*

☐ I have no impairment / I have no illness

☐ I can perform my work, but I have complaints

☐ I'm sometimes forced to work slower or to change my working methods

☐ I'm often forced to work slower or to change my working methods

☐ Because of my illness, I'm only able to work part-time

☐ I can no longer work at all

3. If you think of your current health status and your occupational performance: how do you envision your professional future?
*Please tick the most applicable option only.*

I think that in the next 5 years I'll probably…

☐ …have no severe health impairments that will hinder me from performing at my work place

☐ … be able to continue my profession, but I will have to change my work place due to health impairments

☐ … not be able to continue my profession due to health impairments

☐ … not be able to work at all

## Mental Health

4. In the last 4 weeks, how often did you feel impaired by the following complaints?
*Please put a cross in each line.*

| | not at all | on single days | on more than half of the days | Almost every day |
|---|---|---|---|---|
| Little interest or joy in your activities | ☐ | ☐ | ☐ | ☐ |
| Dejection, melancholy, hopelessness | ☐ | ☐ | ☐ | ☐ |
| Nervousness, anxiousness, tension | ☐ | ☐ | ☐ | ☐ |
| Not being able to stop or control worries | ☐ | ☐ | ☐ | ☐ |

## Functional Ability

5. In the last 4 weeks, to what degree were you able to perform the following?
*Please put a cross in each line.*

| | without any problems | little difficulties | great difficulties | impossible |
|---|---|---|---|---|
| Carry a heavy object over 5 meters | ☐ | ☐ | ☐ | ☐ |
| Perform physical work for 30 minutes | ☐ | ☐ | ☐ | ☐ |
| Stand for 30 minutes without a break | ☐ | ☐ | ☐ | ☐ |
| Climbing a staircase over 3 floors | ☐ | ☐ | ☐ | ☐ |

## Coping Behavior

6. In the last 4 weeks, how well were you able to cope with the following stress situations? *Please put a cross in each line.*

| | very good | better | worse | not at all |
|---|---|---|---|---|
| Time and appointment pressure | ☐ | ☐ | ☐ | ☐ |
| Conflicts, emotionally stressful situations | ☐ | ☐ | ☐ | ☐ |
| High responsibility, high demands | ☐ | ☐ | ☐ | ☐ |
| Physical load | ☐ | ☐ | ☐ | ☐ |

## Sports and Exercise Behavior

7. In the last 4 weeks, how many hours did you perform the following physical activities? *Please put a cross in each line.*

| | 2 hours or more | 1 hour up to 2 hours | up to 1 hour | not at all |
|---|---|---|---|---|
| Physical exercise | ☐ | ☐ | ☐ | ☐ |
| Cycling or running | ☐ | ☐ | ☐ | ☐ |
| Physical activity at work | ☐ | ☐ | ☐ | ☐ |
| Activities at home | ☐ | ☐ | ☐ | ☐ |
